# Impaired Personality Functioning in Children and Adolescents Assessed with the *LoPF-Q 6-18 PR* in Parent-Report and Convergence with Maladaptive Personality Traits and Personality Structure in School and Clinic Samples

**DOI:** 10.3390/children10071186

**Published:** 2023-07-08

**Authors:** Gresa Mazreku, Marc Birkhölzer, Sefa Cosgun, André Kerber, Klaus Schmeck, Kirstin Goth

**Affiliations:** 1 Department of Forensic Child and Adolescent Psychiatry, Psychiatric University Hospitals Basel, 4002 Basel, Switzerlandkirstin.goth@uks.eu (K.G.); 2 Private Clinic, 34740 Istanbul, Turkey; 3 Department of Clinical Psychological Intervention, Freie Universität Berlin, 14195 Berlin, Germany; 4 Department of Clinical Research, Medical Faculty, University of Basel, 4001 Basel, Switzerland; 5 Department of Child and Adolescent Psychiatry, University Clinics Saarland (UKS), 66421 Homburg, Germany

**Keywords:** personality disorder, functioning, maladaptive traits, structure, Criterion A, Criterion B, children, adolescents, parent report, assessment

## Abstract

To investigate if the Personality Disorder (PD) severity concept (Criterion A) of the ICD-11 and DSM-5 AMPD is applicable to children and adolescents, following the ICD-11 lifespan perspective of mental disorders, age-specific and informant-adapted assessment tools are needed. The *LoPF-Q 6-18 PR* (Levels of Personality Functioning Questionnaire Parent Rating) was developed to assess Impaired Personality Functioning (IPF) in children aged 6–18 in parent-reported form. It is based on the established self-report questionnaire *LoPF-Q 12-18*. Psychometric properties were investigated in a German-speaking clinical and school sample containing 599 subjects. The final 36-item version of *LoPF-Q 6-18 PR* showed good scale reliabilities with 0.96 for the total scale IPF and 0.90-0.87 for the domain scales Identity, Self-direction, Empathy, and Intimacy/Attachment and an acceptable model fit in a hierarchical CFA with CFI = 0.936, RMSEA = 0.078, and SRMR = 0.068. The total score discriminated significantly and with large effect sizes between the school population and (a) adolescent PD patients (*d* = 2.7 standard deviations) and (b) the younger patients (6–11-year-olds) with internalizing and externalizing disorders (*d* = 2.2 standard deviations). Informant agreement between parent and self-report was good at 0.47. Good construct validity can be assumed given sound covariation with related measures of psychopathology (*CBCL 4-18*, *STiP-5.1*, *OPD-CA2-SQ PR*) and maladaptive traits (*PID5BF+ M CA IRF*) in line with theory and matching the result patterns obtained in older samples in self-report. The results suggest that parent-reported assessments of IPF and maladaptive traits are equivalent to self-reported measures for Criterion A and B. Assessing IPF as early as age six might be a valuable step to foster early detection of PD, or maladaptive personality development, respectively individuals at risk.

## 1. Introduction

As stated in the World Health Organization’s 2019 report [1], the ICD-11 guidelines for diagnosing Personality Disorders (PD) have undergone significant changes. They have introduced a unifying linear severity dimension, which ranges from ‘no personality pathology’ to ‘personality difficulties’ and progresses to ‘mild’, ‘moderate’, or ‘severe’ personality disorder. This replaces the previous categorical diagnosis of several distinct PD types. The severity of PD is evaluated based on impairments in the basic domains of personality functioning: aspects of the self (e.g., Identity, capacity for Self-direction) and/or problems in interpersonal functioning (e.g., close relationships, understanding others’ perspectives). This corresponds closely to the Alternative Model of Personality Disorders (AMPD) in the DSM-5, with an overall measure of PD severity (Criterion A) based on impairments in self (Identity, Self-direction) and interpersonal (Empathy, Intimacy) functioning [2]. Similarly, Operationalized Psychodynamic Diagnostics [3] uses a severity model of a patient’s structural impairment with four dimensions: Control, Identity, Interpersonality, and Attachment.

Another significant change promoted by the ICD-11 is the introduction of the ‘lifespan approach’ [4]. This concept suggests that psychiatric disorders should be understood with respect to early signs and precursors in childhood, reflecting a shift in the conceptualization of these disorders [5]. Consequently, problematic developments related to impairments in personality functioning could, in theory, be identified at a very early stage. There are no psychiatric disorders that only occur in special developmental phases [6]. However, according to the concept of heterotypic continuity, the phenotype of disorders changes over time [7]. Therefore, age-appropriate diagnostic criteria and assessment instruments have to be developed. This is especially true for disorders of personality, where developmental considerations have led to the reluctance to study the disorder in age groups <18 years [8].

PDs in early to mid-adolescence are subjects of growing research interest, as they are among the most severe mental health problems [9,10,11,12]. PDs are associated with poor psychosocial and physical health, increased psychiatric comorbidity, and high societal costs [10]. As such, early diagnosis of PDs is crucial, given the potential for pervasive impairment across all areas of life [13]. In a longitudinal study, Wertz et al. [13] examined the relationship between Borderline PD (BPD) symptoms in 12-year-olds and various life impairments at age 18. Their findings suggested that the top 5% of individuals with the most severe BPD symptoms had a significantly higher risk of mental disorders, suicide attempts, professional failure, lower life satisfaction, and a higher frequency of criminal activities. Consequently, early and accurate detection of individuals suffering from PDs is of paramount importance to ensure they receive effective, empirically based treatments and, consequently, to prevent impairments in adulthood.

While Criterion A of the DSM-5 AMPD defines the severity of the PD, Criterion B specifies the PD style by assessing an individual’s profile of maladaptive personality traits [2]. Criterion B includes five overarching maladaptive trait domains that can be aligned with the Big Five Trait concept: Negative Affectivity (vs. Emotional Stability), Detachment (vs. Extraversion), Antagonism (vs. Agreeableness), Disinhibition (vs. Conscientiousness), and Psychoticism (vs. Lucidity) [14]. Each of these trait domains is composed of a total of twenty-five trait facets, which can be assessed using the Personality Inventory for DSM-5 (*PID*-*5* [15]). Bach et al. [16] demonstrated, based on clinical data of over 200 psychiatric patients, that the DSM-5 traits are strongly related to the categorical classification of PDs. This indicates that the new trait model reliably captures a substantial amount of information of the former classification.

In ICD-11, the dimensional conceptualization of PD using a two-fold assessment of severity and style was largely adopted, with some modifications concerning the assessment of PD traits/styles. Although in the ICD-11 a style description of PD in terms of Criterion B is not mandatory, five maladaptive trait domains, namely Negative Affectivity, Detachment, Dissociality, Disinhibition, and Anankastia, are included for this purpose [1]. Anankastia is defined by perfectionism, orderliness, and rigidity and is specific to ICD-11, while the *PID*-*5* trait Psychoticism is not reflected in ICD-11 [17]. Oltmanns and Widiger [18] have studied the correlations between the ICD-11 and the DSM-5 maladaptive trait domains. They found strong correlations between DSM-5 and ICD-11 Negative Affectivity, Detachment, and Disinhibition. Furthermore, there was a strong correlation between the DSM-5 domain Antagonism and the ICD-11 domain Dissociality. Based on findings by Bach et al. [19] and Sellbom et al. [20], which demonstrated the applicability of the DSM-5 AMPD maladaptive traits to the ICD-11 PD domains, the *PID5BF+ M* was developed [16,21]. It is a brief form of the *PID*-*5,* additionally capturing the ICD-11 domain Anankastia, assessing 18 out of the 25 facets of the *PID*-*5* using only 36 items [21]. Initially developed for adults, some of the items have been slightly reformulated and simplified to be appropriate for adolescent self-report from 12 years of age (*PID5BF+ M A* [22]) and for parent-report for children 6 years of age and older (*PID5BF+ M CA IRF* [23]). In this study, this age-adapted version for parent report was used for the first time and showed good psychometric properties in this sample (see Section 2). 

A recent analysis of data of the US National Comorbidity Survey (*N* = 9282) using structural equation modeling revealed that BPD symptoms such as identity diffusion, emotion regulation, and interpersonal problems explain a large amount of variance of a general factor of psychopathology [24]. More recent research supports the central role of PF in understanding psychopathological symptoms. Generally, PF seems to be associated with well-studied transdiagnostic constructs such as interpersonal problems, insecure attachment styles, emotion regulation difficulties, pathological beliefs, and maladaptive schemas [25,26]. Furthermore, the *PID*-*5* and its shorter forms can be used to assess the Hierarchical Taxonomy of Psychopathology (*HiToP*), which is a dimensional approach for the classification of a wide range of psychiatric problems [27].

To enable investigations on the applicability and clinical utility of the PD severity concept in children and young adolescents, age-specific and informant-adapted reliable and clinically valid assessment tools are needed and have to be developed. It is important to note that the diagnosis of mental illnesses in children and adolescents should be based on multiple diagnostic components that complement each other [28]. In addition to clinical assessments and self-reports, caregiver-reports should also be considered for a valid diagnosis of mental illnesses in children and adolescents, as they have a deep understanding of the child’s development over time.

Parent reports are considered the standard method for assessing symptoms of mental illnesses in children and adolescents in clinical and research settings. The most commonly used measures for assessing overall psychopathology in parent report include the Child Behavior Checklist (CBCL 4-18 [29]), as well as the parent versions of the Strength and Difficulties Questionnaire (SDQ-P [30]) and the Behavior Assessment System for Children (BASC-3 [31]). These inventories can be used as informant-based assessments from preschool age to adolescence. Parent reports are also widely used concerning general characteristics of children, such as temperament and personality traits. Some commonly employed parent reports include the Child Behavior Questionnaire (CBQ [32]), the Inventory for Child Individual Differences (ICID [33]), the Behavioral Inhibition System/Behavioral Activation System Scales (BIS/BAS-Scales Parent Report [34]), the Emotionality-Activity-Sociability Surveys (EAS Parent Report [35]), the Hierarchical Personality Inventory for Children (HiPIC [36]), and the respective parent versions of the Junior Temperament and Character Inventory for preschoolers, primary schoolers, and adolescents [37,38,39].

### Development of the Age-Specific Assessment Tool LoPF-Q 6-18 PR

Over the last decade, several self-report questionnaires specifically developed for adolescents from 12 years up were developed and introduced by the University Hospitals Basel (UPK, Switzerland) research group specialized in the development of diagnostic assessment tools for younger ages. These questionnaires include *AIDA* (Assessment of Identity Development in Adolescence [40]), *LoPF*-*Q 12-18* (Levels of Personality Functioning Questionnaire [41]), *OPD*-*CA2*-*SQ* (Operationalized Psychodynamic Diagnosis in Children and Adolescents—Structure Questionnaire [42]), and the *DSQ*-*22*-*A* (Defense Style Questionnaire—22-item version for Adolescents [43]). Several culture-adapted versions of the tests with nation-specific population norms are established, e.g., *LoPF*-*Q 12-18* in English [44], in Spanish [45], and in Turkish [46,47].

The general focus of test construction in this research group is clinical validity in terms of providing sound and practical support in diagnostic decision-making. Item formulations were developed in expert teams of clinicians from the field and methodologists specialized in test construction and validation. Each item is supposed to (a) display a reasonable linear variation from “healthy” to “impaired”, (b)be unambiguous and (c) avoid typical gender bias and social desirability. Furthermore, the items should display concrete problem behavior in order to deal with the issue of constrained evaluation on scales of impairment. The final item sets are progressively deduced using statistical item selection in a series of beta, pilot, and main tests, following the ITC guidelines for test construction [48]. In all assessments, PD patients or at least subjects from clinical samples with pronounced emotional and behavioral problems are included in the samples to cover the full target group and distribution of the scales. Moreover, the empirical item selection is not only based on the classical criteria of psychometric properties (e.g., itemtotal correlation) but also considers the effect size of the differentiation between healthy and impaired samples (e.g., patients diagnosed with PD) on item level [49].

The four *LoPF*-*Q 12-18* domains of impaired personality functioning, “Identity, Self-direction, Empathy/Prosociality, and Intimacy/Attachment,” are designed as distinct units to inform differential diagnoses and therapy planning while adding up to the joint construct of PD severity to enable a global evaluation of “Impaired Personality Functioning” using a total score. In a CFA at item level, a corresponding bifactor model with a strong general factor and four specific factors showed a good fit [50]. The *LoPF*-*Q 12-18* total score “Impaired Personality Functioning” differed between adolescents from a school population and a subsample of *N* = 96 patients diagnosed with PD. This difference was highly significant, with a large *d* = 2.1 standard deviations effect size. The four scales, Identity, Self-direction, Empathy, and Intimacy, showed effect sizes *d* of 2.0, 1.7, 0.9, and 2.2, respectively [49]. The differentiation between healthy and impaired groups even increased when considering the theoretically corresponding PD type. Impairments in the aspect of self-functioning “Identity” showed the highest impact in BPD patients, whileimpairments in Self-direction were strongest in Anxious-avoidant PD, and impairments in Empathy/Prosociality were strongest in Antisocial or Narcissistic PD.

**Figure 1:**
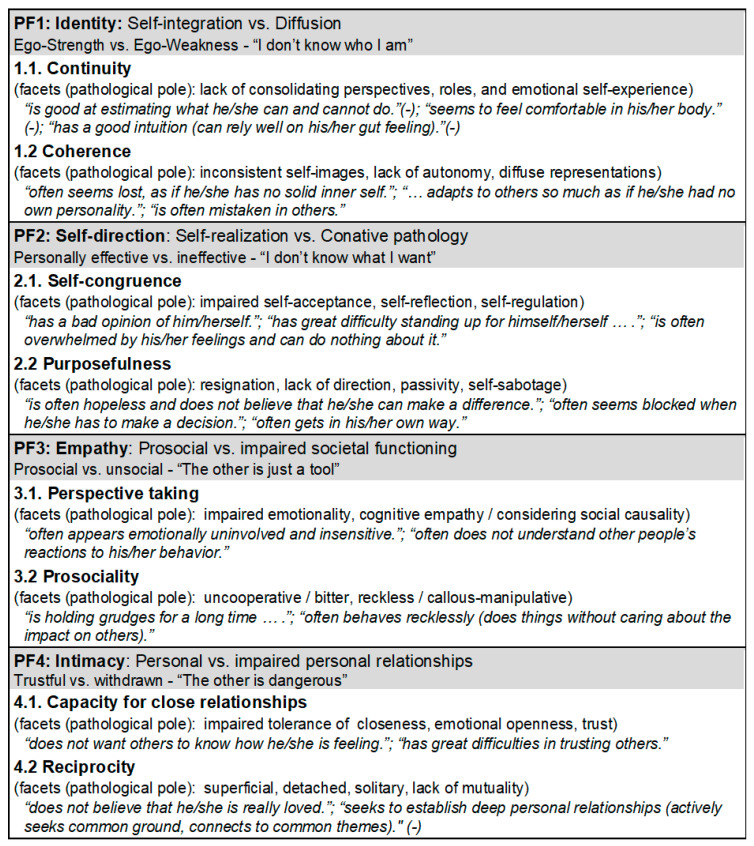
*LoPF-Q 6-18 PR* model to structure the four domains of Impaired Personality Functioning (IPFs) into scales, subscales, and facets. The pathological poles of the facets and marker items are given to clarify the integrated aspects of impairment. (-) = Item formulation represents non-pathological functioning.

In order to track the course of impaired personality development from childhood to adolescence on equivalent dimensions, the established self-report questionnaire *LoPF*-*Q 12-18* for adolescents from 12 years up was used as the basis to develop an age- and informant-adapted test version for measuring the severity of Impairments in Personality Functioning (IPF) in children and adolescents aged 6–18 in parent-report (*LoPF-Q 6-18 PR* [51]). From the 97 items, those that (a) showed the strongest clinical validity and best reliability, (b) showed potential to be adapted to a younger age, and (c) seemed well amenable to informant assessment were selected and re-formulated (e.g., for Identity: “I feel comfortable in my body” (self-report) was reformulated to “seems to feel comfortable in his/her body” (parent-report)). Additional items were formulated to potentially represent all scales and subscales of the youth version for those aspects that needed stronger age-adaption to fit the specific life circumstances (e.g., for Self-direction: “I often feel that I am a victim of my life circumstances” (self-report for adolescents) was changed to “is often hopeless and does not believe that he/she can make a difference” (parent-report) to avoid the term “victim” because parents would blame themselves with that and children between 6 and 12 do not have much freedom of choice concerning their life circumstances). In beta tests and a pilot test with *N* = 80 parents, a large item pool was developed from which the final version of the questionnaire was empirically selected. Figure 1 shows the full LoPF-Q model of scales, subscales, and aspects that are equivalent for all age and informant versions, enlarged with item examples from the parent version.

In the present study, scale reliability, factorial validity, and clinical validity of the *LoPF*-*Q 6-18 PR* in terms of (a) discrimination between school and clinic samples and (b) the relation to other measures of psychopathology (*CBCL 4-18*, *STiP*-*5.1* interview) are examined. Moreover, the correlation between self-reported (*LoPF*-*Q 12-18*, original and short version) and parent-reported (*LoPF*-*Q 6-18 PR*) Impaired Personality Functioning (IPF) and the relations to the constructs “maladaptive traits” (*PID5BF+ M CA IRF*) and “personality structure” (*OPD*-*CA2*-*SQ PR*) are investigated in order to compare the result patterns between those related constructs in parent-report to the known patterns in self-report and older ages.

## 2. Materials and Methods

### 2.1. Participants and Procedure

The total sample consisted of *N* = 599 children and adolescents (*N* = 343 from a representative school sample, *N* = 256 from a clinical sample with diagnosed mental disorders). The sample included two age groups: *N* = 270 children aged 6 to 11 years (only parent or caregiver reports were obtained) and *N* = 329 adolescents and young adults aged 12 to 26 years (parallel self-reports were obtained). Inclusion criteria required sufficient skills in the German language to master the written task. As all age-adapted questionnaires can be used up to young adulthood in principle, participants older than 18 (*N* = 9 participants) were not excluded as they were part of the assessed school classes or clinical units. The distributive characteristics of the sample support the assumption that a sufficiently representative sample was achieved by the survey design in total (see Table 1).

The school sample was collected at multiple schools and youth sports clubs in Switzerland (urban: 76%, rural: 24%). Due to then-effective COVID restrictions, teachers or trainers carried out study information and distribution of test materials. The clinical sample was collected at inpatient and outpatient departments of child and adolescent clinics in Switzerland, Austria, and Germany (Basel, Innsbruck, Kassel, Saarland, and Berlin), as well as at the Admission Center Basel (AH Basel) and the child and adolescent psychiatric practice of R. Weissensteiner in Vienna. Written consent was obtained from the parents. Adolescents aged 14 and older had to consent separately from their parents. Diagnostics of Axis-I and personality disorders (PD) were conducted in accordance with guidelines by trained professionals. PD Patients were assigned to the PD group independently of their Axis-I diagnosis. 

### 2.2. Measures

#### 2.2.1. Adolescents

The *LoPF*-*Q 12-18 German version* (Level of Personality Functioning Questionnaire; [49]) is a questionnaire for measuring Impairments in Personality Functioning (IPF) in adolescents aged 12-18 (±2 years, up to young adulthood) in self-report. It contains 97 items with a 5-step answering format (0 = no to 4 = yes). It provides a total score IPF, the four domain scores Identity, Self-direction, Empathy/Prosociality, and Intimacy/Attachment, and each two subscales. A higher score indicates a higher impairment. Good scale reliabilities (total scale: 0.97, primary scales: 0.87 to 0.92, subscales: 0.76 to 0.91) and good factorial validity were obtained [50]. The test showed good clinical validity in terms of discrimination between general populations and PD patients, with a large effect size of *d* = 2.1 standard deviations (see above) [49]. The test is available free of charge for research purposes. It can be used with nation-specific population norms obtained in *N* = 351 adolescents from three schools in the Basel area in Switzerland.

On a trial basis, a special short version, *LoPF*-*Q 12-18 Short German version,* of the original version for adolescents was derived in this study. This matches the parent version on the item level as closely as possible: both versions have 36 items representing exactly the same scales, subscales, and aspects. This short version combines the 20 items of the “Screener version” that was created using Ant Colony Optimization and CFA [50] plus an additional 16 items with excellent psychometric properties to (a) enable the use of subscales in addition to the primary scale level and (b) match the parent report version. However, this must be regarded as a research version; psychometric properties have to be obtained in new samples. All research versions can be requested from the last author of this article.

The *MINI*-*KID German version* (Mini-International Neuropsychiatric Interview for Children and Adolescents (MINI-KID [52])) is a short standardized interview used to diagnose Axis I disorders in children and adolescents, based on the criteria of the DSM-IV [53] and ICD-10 [54]. It is an extension of the Mini-International Neuropsychiatric Interview (MINI [55]) for adults and consists of diagnostic modules, each initiated by two to four screening questions. If a screening result is positive, additional questions about specific disorders are asked. The MINI-KID provides reliable and valid psychiatric diagnoses with a short administration time. 

The *SCID*-*II German version* (Structured Clinical Interview for DSM-IV Axis II Personality Disorders [56]) is a semi-structured clinical interview used to diagnose PDs according to the DSM-IV classification system. The interview allows for the assessment of 12 PD types (Avoidant, Dependent, Obsessive-compulsive, Negativistic, Depressive, Paranoid, Schizoid, Histrionic, Narcissistic, Borderline, and Antisocial PD) and an unspecified category (“not otherwise specified”). The SCID-II has been well-established and adequately validated for use in adults [57]. Additionally, the instrument has been successfully used in research projects with adolescents with only a few adjustments [49,58,59]. For the current study, the interview questions regarding Antisocial PD were adapted to the adolescents’ life world (e.g., the questions concerning driving without a license or being unemployed had to be adapted).

The *STiP*-*5.1 German version* (Semi-Structured Interview for Personality Functioning DSM-5 [60]) is a semistructured interview developed and validated in the Netherlands for assessing personality functioning based on DSM-5 criteria. It measures four personality functions: Identity, Self-direction, Empathy, and Intimacy, each consisting of three facets. The Interviewer rates the interviewed on a 5-point scale (0 = no/minor impairment to 4 = extreme impairment) on these 12 facets. The STiP-5.1 has high inter-rater reliability, with ICCs ranging from 0.79 to 0.92 for the 12 facets and 0.93 for the overall score. The clinical group had significantly higher impairments in personality functioning than the healthy control group. Although the STiP-5.1 was not specifically developed for use with adolescents, it has been successfully used with a sample of 12 to 17-year-old adolescents [61]. The German translation of STiP-5.1 has also been successfully validated [62].

#### 2.2.2. Parents

The *LoPF*-*Q 6-18 PR German version* (Levels of Personality Functioning Questionnaire Parent Report [51]) is a questionnaire for measuring the severity of Impairments in Personality Functioning (IPF) in children and adolescents aged 6–18 in parent-report. It consists of 36 items answered on a 5-step Likert scale (0 = no to 4 = yes). Parallel to the established self-report version for adolescents, it provides a total score IPF, the four domain scores Identity, Self-direction, Empathy/Prosociality, and Intimacy/Attachment, and each two subscales. A higher score indicates a stronger impairment. The test is validated in the current study; for psychometric properties, see the Results section; for details on development, see the Introduction.

The *OPD*-*CA2*-*SQ PR German version* (Operationalized Psychodynamic Diagnosis in Children and Adolescents—Structure Questionnaire Parent Report [63]) is a questionnaire for measuring Impairments in Personality Structure (IPS) in children and adolescents aged 6–18 in parent-report. It comprises 38 items with a 5-step answer format (0 = no to 4 = yes). Parallel to the established self-report version for adolescents, it provides a total “Structural Impairment” score and the four domain scores of Control, Identity, Interpersonality, and Attachment. Scale reliabilities Cronbach’s Alpha in the current sample was 0.96 for the total score and 0.89, 0.88, 0.87, and 0.83 for the four domain scores. Informant agreement between parent report and self-report (OPD-CA2-SQ) was 0.39 for the total score and 0.35, 0.43, 0.34, and 0.34 for the four domains of structural impairment, respectively. The OPD-CA2-SQ PR showed clinical validity, with the total score differentiating between adolescents from a general population and a subsample of *N* = 38 patients diagnosed with PD (*SCID*-*II*) at a highly significant level and with a large effect size of *d* = 2.5 standard deviations. The result pattern was nearly identical when contrasting children aged 6–11 from the school population and the patient sample of *N* = 57 patients aged 6–11 (with mostly comorbid externalizing and internalizing problems) with a large effect size of *d* = 2.3 standard deviations.

The *PID5BF+ M CA IRF German version* [23] is a questionnaire designed to be completed by parents or close caregivers to assess maladaptive traits based on the PID-5 concept in children and adolescents aged 6 to 18 years (up to young adulthood). The PID-5 is the official rating scale of the American Psychiatric Association for the assessment of maladaptive personality traits according to criterion B of the alternative model for personality disorders in Section III of the DSM-5 [2]. The PID5BF+ is suitable for assessing maladaptive personality traits according to both DSM-5 and ICD-11. The PID5BF+ was developed using ant colony optimization algorithms. The validity of the model and the assessment tool could be confirmed in large German- and English-speaking samples [21]. The PID5BF+ M differs only in the definition of the Anankastia domain. The validity of this modified version could be ascertained in samples of 15 different countries [16]. The PID5BF+ M CA IRF was developed as a modified version for parent report in collaboration with the original authors, Kerber, Krueger, Bach, and Zimmermann. The inventory measures the six personality trait dimensions of Negative Affectivity, Detachment, Antagonism, Disinhibition, Anankastia, and Psychoticism using 36 items on a 4-point response scale (0 = Very false to 3 = Very true). In a sample from the current study of *N* = 336 non-clinical and *N* = 116 clinical cases, the PID5BF+ M CA IRF showed good scale reliabilities between 0.79 and 0.89 for the six scales. Detailed analyses of the PID5BF+ M adolescent versions are in progress and will be published elsewhere.

The *CBCL 4-18 German version* (Child Behavior Checklist [64]) is used to assess competencies, emotional problems, and behavioral problems of children and adolescents aged 4 to 18 years from the parents′ perspective. The CBCL 4-18 is part of the Child Behavior Checklist inventory family [29], whose clinical screening inventories are used internationally and validated well. The questionnaire consists of 120 statement items, rated on a 3-point scale (0 = Not true, 2 = Somewhat true, or 3 = Very true). These items are summarized into the eight syndrome scales Social Withdrawal, Somatic Complaints, Anxiety/Depression, Social Problems, Thought Problems, Attention Problems, Rule-Breaking Behavior, and Aggressive Behavior. In this study, the aggregated second-order problem scales “Internalizing” (Social Withdrawal, Somatic Complaints, Anxiety/Depression), “Externalizing” (Rule-Breaking Behavior, Aggressive Behavior), and “Total Problems” (all eight syndrome scales) will be used, which have good scale reliabilities (Cronbach’s Alpha between 0.85 and 0.95).

### 2.3. Data Analytic Plan

Data analysis was conducted using SPSS version 26 and R version 4.2.2. Confirmatory factor analysis (CFA) was performed using the R package ‘lavaan’ [65], and scale reliabilities (Omega hierarchical [66]) were estimated using the R package ‘semTools’ [67].

Scale reliabilities were evaluated by (a) Cronbach’s Alpha and (b) McDonald’s Omega hierarchical (ωh [66]). Cronbach’s Alpha is a widely used and accepted measure of internal consistency. Yet, it assumes that all items in a test are equally correlated, a condition that is often not met in practice. On the other hand, McDonald’s Omega hierarchical is considered a more robust measure of reliability, as it does not assume equal correlations among items and is more appropriate for multifactorial constructs [66]. Furthermore, Omega hierarchical specifically assesses the proportion of total score variance attributable to a general factor, which is particularly relevant in hierarchical models such as those used in this study [68,69]. Therefore, using both of these measures allowed to provide a more comprehensive and robust estimation of the reliability of the scales. Scale reliabilities were supposed to exceed 0.80 at total scale level, 0.70 at primary scale level, and 0.60 at subscale level as appropriate for heterogeneous contents, while homogeneity coefficients >0.80 would be very good and >0.90 excellent. Aspects of construct validity were tested with product-moment correlations at the 1% significance level and with criteria for effect size: *r* > 0.10 small, >0.30 medium, and >0.50 strong. For correlational informant agreement between self-report and informant-report (different informant and different assessment tool), r coefficients >0.40 were considered strong, following Carlson, Vazire, and Oltmanns [70]. 

Matching the statistical procedure used for the German *LoPF*-*Q 12-18 Screener* [50], factorial validity was investigated using CFA with a hierarchical model with four first-order factors to represent the domains and a secondary higher-order factor to represent Impaired Personality Functioning (IPF). In this study, the Weighted Least Squares Mean-Variance (WLSMV) estimator was used as it allows for non-normal data and missing values while providing accurate model fit indices [71]. Comparative Fit Index (CFI), Root Mean Squared Error of Approximation (RMSEA), and Standardized Root Mean Square Residual (SRMR) were used as fit indices (fit criteria: CFI > 0.900 acceptable and CFI > 0.950 good, RMSEA < 0.050 good and < 0.080 acceptable, SRMR < 0.080) [72]. 

Group comparisons were performed with the raw scores using MANOVA (multivariate analysis of variance). Score differences were examined not only concerning significance (1% level) but concerning effect size (“Cohen’s d”), taking into consideration big differences in sample size and for a better intuitive interpretation of the results, as *d* = 1 corresponds to the familiar unit “1 standard deviation” to describe a difference [73]. Cohen’s d should be higher than 0.80 to avoid over-interpretation of statistically significant results.

## 3. Results

### 3.1. Descriptive Analyses

The characteristics of the full sample, such as gender distributions, age ranges, and diagnostic information, are presented in Table 1. In the clinical sample, 14.8% of the individuals were diagnosed with Personality disorder (PD), 34.4% with internalizing disorders (e.g., anxiety disorders, depression, phobias, obsessive-compulsive disorder), 14.8% with externalizing disorders (e.g., attention-deficit and hyperactivity disorder, conduct disorder, substance-related disorders), and 27.3% showed problems from both areas and were assigned to the group “comorbid” (e.g., post-traumatic stress disorder, adjustment disorders). The most frequent PD diagnoses in the clinic sample were Antisocial PD (47.4%) and Borderline PD (21.1%). Formal PD diagnoses were obtained in the adolescent patient sample (12–18 years) only, as formal PD diagnostics at younger ages are most probably not adequate. According to the *N* = 343 parent-rated *CBCL 4-18*, the school sample (covering the full age range from 6 years up) seems sufficiently representative, with 3% of cases strongly above the average denoting relevant emotional and behavioral problems.

Data from the school sample demonstrated a sufficient normal distribution of the *LoPF*-*Q 6-18 PR* scores on the total score, primary scale, and subscale level, with skewness and kurtosis displayed at values around ׀1׀. No meaningful score differences were obtained between girls and boys. They were significant for the total scale only at the 5% level (criteria: 1% level) and not significant for most scales and subscales. Moreover, no difference reached a relevant effect size (criterion: strong effect size). The differences between younger and older children and adolescents were not significant at all scale levels. 

### 3.2. Reliability and Correlational Informant Agreement between Parent-Report and Self-Report

The *LoPF-Q 6-18 PR* demonstrated good scale reliabilities using Cronbach’s Alpha (0.96 for the total scale, 0.87, 0.90, 0.90, and 0.88 for the primary scales; see Table 2), as well as using McDonald’s Omega hierarchical (0.99 for the total scale, 0.96, 0.94, 0.88, and 0.90 for the primary scales). The four primary scales showed medium-to-high phenotypic intercorrelations between 0.71 and 0.83.

The correlational informant agreement between the parent-rated *LoPF-Q 6-18 PR* and the self-rated *LoPF-Q 12-18* was highly significant (*p* = 0.000) for all scales and was strong for the total score (0.47) and three of the four domains: Identity (0.43), Self-direction (0.47), and Intimacy (0.50). The correlation between parent- and self-rated Empathy (0.36) was slightly below the criterion (>0.40), but it met the set criteria of *r* > 0.40 and *p* < 0.05. Only the Empathy domain demonstrated a lower correlation, with *r* = 0.36, but the correlation obtained a high significance level nonetheless (see Table 2). 

### 3.3. Factorial Validity

**Figure 2:**
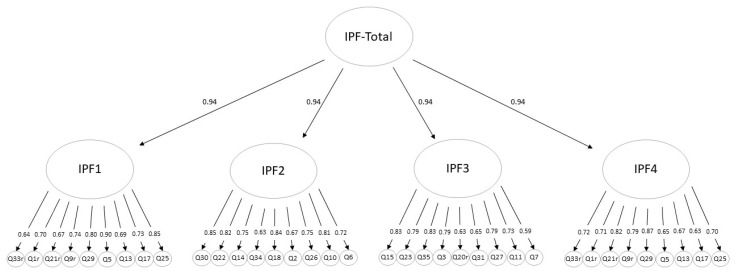
Factor loadings. A hierarchical model with four first-order factors to represent the domains (IPF1-4) and a secondary higher-order factor to represent impaired personality functioning (IPF-Total).

A hierarchical model was tested by matching the theoretical assumptions and the statistical approach elaborated by Zimmermann et al. [50]. This model included four first-order factors representing the four domains of personality functioning and a higher-order factor representing Impaired Personality Functioning (IPF). The model also assumed equal factor loadings on the general factor. The global fit index was significant, χ^2^ (593, *N* = 599) = 2363.04, *p* < 0.001. The fit indices from the CFA (CFI = 0.936, RMSEA = 0.078, and SRMR = 0.068) indicate an acceptable fit of this model to the data, matching all criteria (see Section 2.3). All 36 items showed high loadings on the assigned domain of personality functioning, and all four domains demonstrated high loadings on the joint higher-order factor. Factor loadings are shown in Figure 2.

### 3.4. Covariation with PD Pathology

The *LoPF*-*Q 6-18 PR* total score differed between adolescents aged 12-18 from the school sample and a PD patient sample of *N* = 38. This was very significant, with a large effect size of *d* = 2.8 standard deviations. All four domain scales (Identity, Self-direction, Empathy, and Intimacy) showed strong discrimination between the school and the PD sample with large effect sizes (*d* ranged from 2.0 to 3.1; see Table 3).

For the age group 6–11, this comparison to investigate clinical validity was impossible because PD diagnoses most probably do not apply to children. Thus, the full clinical sample of 6–11-year-olds was compared with the school sample in the younger age group. The differences in scores were nearly in the same range as for the adolescents. All scores differed highly significantly, with large effect sizes (*d*) ranging from 1.8 to 2.2 (see Table 4).

### 3.5. Convergence with Broader Psychopathology, Maladaptive Traits, and Personality Structure

The correlation between parent-rated Impairment of Personality Functioning (IPF) using the *LoPF*-*Q 6-18 PR* questionnaire and the Level of Personality Functioning using the *STiP*-*5.1* interview for adolescents was significant. It ranged from moderate to strong for the total score and the domains of intrapersonal functioning Identity and Self-direction. However, the scales for interpersonal personality functioning Empathy and Intimacy showed no significant correlations (see Table 5).

The conceptually related parent-report questionnaires *LoPF*-*Q 6-18 PR* (assessing impairments in personality functioning) and *OPD*-*CA2*-*SQ PR* (assessing impairments in personality structure) showed very strong positive and highly significant correlations on all scale levels. The two questionnaires exhibited a correlation of 0.96 between their total scores, indicating a high degree of agreement in capturing structural or functional impairments. 

The convergence between the *LoPF*-*Q 6-18 PR* scales and the *PID5BF+ M CA IRF* scales, used to assess maladaptive traits, was highly significant. Strong correlations, >0.50, were observed between the IPF total score and the maladaptive traits. Specifically, there were strong correlations with Negative Affectivity (0.62), Detachment (0.78), Antagonism (0.65), Disinhibition (0.66), and Psychoticism (0.69). However, the maladaptive trait Anankastia showed only a small correlation of 0.25 with the total score IPF and 0.19 to 0.28 with the IPF domain scales. 

Broader psychopathology in children and adolescents assessed in parent-report using the *CBCL 4-18* showed strong correlations with impairments in functioning assessed with the *LoPF*-*Q 6-18 PR*. Correlations were highly significant and strong, with coefficients of 0.78 between the two total scores. The IPF total score was correlated to 0.70 with CBCL internalizing problems and 0.68 with CBCL externalizing behaviors. The only correlation that reached a medium effect size was between IPF Intimacy and externalizing behavior. 

### 3.6. Short Version of the LoPF-Q 12-18

The original *LoPF*-*Q 12-18* for adolescent self-report contains 97 items. Many researchers wish for shorter research versions. Thus, in the current study, a first attempt was made to investigate the psychometric properties of a short version that is parallel in length to the parent-report version. 

A shortened version with 36 items (taken out of the assessed 97-item version) showed scale reliabilities in the current sample. The Cronbach’s Alpha coefficients were 0.96 on total and 0.87, 0.90, 0.81, and 0.85 on the primary scale level for Identity, Self-direction, Empathy, and Intimacy, respectively. McDonald’s ωh also showed good scale reliabilities for the total score (0.98) and the primary scales (0.91, 0.94, 0.72, and 0.87, respectively). Informant agreement with the parent-version *LoPF*-*Q 6-18 PR* was significant for all scales, with 0.37 for the total score and 0.31 for Identity, 0.42 for Self-direction, 0.21 for Empathy, and 0.40 for Intimacy. The 36-item *LoPF*-*Q 12-18 Short* showed a good model fit in a hierarchical CFA with four first-order factors and one higher-order factor matching all criteria (CFI = 0.951, RMSEA = 0.060, and SRMR = 0.068). This is the same method used with the 36-item *LoPF*-*Q 6-18 PR*. In terms of a first check of clinical validity, the total score discriminated highly significantly, with an effect size of *d* = 1.1 standard deviations between the school and the adolescent PD patients in the current sample.

## 4. Discussion

This study aimed to provide an age- and informant-adapted measurement tool for assessing Impaired Personality Functioning (IPF) in children and adolescents in parent-report. This was conducted in response to the ICD-11’s call for a lifespan approach and the assertion that, given the appropriate assessment tools, early indicators of psychopathology can be identified even at younger ages [4]. The *LoPF*-*Q 6-18 PR* (Levels of Personality Functioning Questionnaire—Parent Report for 6-18 year olds) was developed to meet this need.

The investigations focused on examining the reliability, factorial validity, and clinical validity of this tool. Specifically, the ability to differentiate between a general school sample and individuals diagnosed with Personality Disorders (PD) was assessed. Moreover, the level of agreement between parent-reported and self-reported scores was evaluated.

Additionally, to understand the measure’s congruent and discriminant validity and to map its position within the nomological network of psychological constructs, the relations with other established measures of psychopathology, such as the Child Behavior Checklist (*CBCL 4-18*) and the Structured Interview for Personality Organization in Children and Adolescents (*STiP*-*5.1* interview), were analyzed. Furthermore, the associations with maladaptive traits as assessed by the Personality Inventory for DSM-5—Brief Form Plus—Child Age—Informant Report Form (*PID5BF+ M CA IRF*) and the personality structure as gauged by the Operationalized Psychodynamic Diagnosis in Children and Adolescents—Structure Questionnaire—Parent Report (*OPD*-*CA2*-*SQ PR*) were examined.

These findings can serve as the basis for a more nuanced understanding of IPF in children and adolescents and contribute to developing effective diagnostic tools and therapeutic interventions. They also highlight the importance of involving multiple informants, including parents, in the assessment process, as this can provide a more comprehensive and accurate picture of a young individual’s personality functioning. All new parent-report versions presented here are available free of charge for research purposes and can be used with T-normation from Switzerland (https://academic-tests.com).

The attempt to adapt the concept of IPF to parent-report for children and adolescents from 6 years up with similar psychometric properties and result patterns compared to adolescent self-report was successful. This applies to the good scale reliability, the acceptable model fit in a hierarchical CFA, and the good construct validity in terms of the scales’ clear references to psychopathology and maladaptive traits. 

Consistent with results from prior studies using adolescent self-report data [50], the parent report version *LoPF*-*Q 6-18 PR* showed an acceptable fit to the data with 36 items representing the four domain scales Identity, Self-direction, Empathy, and Intimacy and the total scale Impaired Personality Functioning (IPF). Scale reliabilities were good on primary and total scale levels concerning Cronbach’s Alpha and McDonald’s Omega. This is very much in line with the diagnostic models of DSM-5 and ICD-11, where IPF is seen as an overarching construct incorporating different domains of impaired psychosocial functioning in general. The current study might be the first to empirically describe the established LPF factor structure in a sample of children from 6 years of age up.

In accordance with the constructional goal to assess IPF in children as a potential early sign or risk factor for PD and the severity thereof (Criterion A), parent-rated IPF was much higher in the clinical sample. This was reflected by higher scores on all scales compared to the school sample. The parent-rated total score IPF discriminated highly significantly, with a huge effect size of *d* = 2.8 standard deviations between the adolescent school sample and the adolescent clinical sample of *N* = 38 with diagnosed Personality Disorders (PD). These findings mirror the results for adolescent self-report [49]. The resulting pattern was nearly identical in the younger age group of 6–11-year-olds, where parent-rated IPF was much higher in the clinical sample. This was reflected by higher scores on all scales compared to the school sample. Additionally, in the group of 6–11-year-olds, the parent-rated total score IPF discriminated highly significantly, with a huge effect size of *d* = 2.2 standard deviations between the school sample and the clinical sample of *N* = 57 patients with mixed diagnoses. Thus, the *LoPF*-*Q 6-18 PR* demonstrated excellent clinical validity in younger and older children, suggesting that the basic IPF concept might be applicable in principle already in primary school age. The high correlations with the syndrome scales of the *CBCL 4-18*, designed to capture axis-1-related impairments in terms of behavioral and emotional problems, further support the clinical validity of parent-rated IPF. These findings indicate that the *LoPF*-*Q 6-18 PR* scores covariate clearly and positively with the pathology-denoting scores of the highly established CBCL. Thus, it can be concluded that LoPF-Q 6-18 PR is at least capturing psychopathology, even if the detailed nomological network of relations of parent-rated IPF needs thorough further investigation. Especially, since no longitudinal data elucidating the further development of children with IPF are available, it is necessary to clarify if IPF in childhood can be considered a precursor or early sign of a PD and to what extent IPF in adolescence shows stability over time in relation to broader psychopathology. IPF in children might simply reflect general psychopathology, matching the high correlation between the *CBCL 4-18* scores and the *LoPF*-*Q 6-18 PR*. On the other hand, this high correlation might indicate that general psychopathology is an expression of underlying IPF at this age. However, further prospective longitudinal studies will be needed to disentangle the association between IPF and general psychopathology in this age group. These studies will provide insights into the developmental pathways of this potential early sign or risk factor concerning PD emergence in adolescence. A large prospective longitudinal study aiming to do just that is on the way, starting in summer 2023 (EARLY-Study, see below). 

The results of the current study also appear to support the convergent validity of the *LoPF*-*Q 6-18 PR*. The scales correlated highly with different measures of psychopathology and with the parent version of PD Criterion B and the ICD-11 maladaptive trait measure *PID5BF+ M CA IRF*. 

Most interestingly, the parent-rated IPF domains showed significant and mostly strong correlations above the criteria with adolescent-rated IPF using the questionnaire *LoPF*-*Q 12-18* (informant agreement of 0.36-0.50) but not for all adolescent-reported IPF using the clinical interview *STiP*-*5.1* (−0.05 for Empathy ns, 0.24 for Intimacy ns). This could be the result of too many essential differences concerning the two assessment tools, such as the change of concept (STiP vs. LoPF), method (interview vs. questionnaire), and informant (parent-report vs. adolescent self-report). Given the very different pattern of intercorrelations on domain levels, it is noteworthy that *STiP*-*5.1* Identity showed its highest correlations with all parent-rated domains of impaired functioning. These correlations were remarkably higher than the intercorrelations between the “same constructs,” such as Self-direction with Self-direction or Intimacy with Intimacy. Therefore, future studies should thoroughly investigate the impact of the change in concepts. Ideally, this investigation should involve the development of a parent version of *StiP*-*5.1*, which can be compared to the parent version of LoPF-Q, to gain a comprehensive understanding of the potential impact and implications of these conceptual changes. On the other hand, to some extent, it can be expected that specifically scores for the intrapersonal domain Empathy differ between parent-rating and self-rating, especially in the given clinical subsample with a high number of patients diagnosed with Antisocial PD (see below). Thus, the congruence between parent-reported and self-reported Empathy in the current sample, which did not show when using the *STiP*-*5.1*, speaks in favor of multi-informant approaches that use assessment tools rooted in the same test family. 

The very strong correlation with the psychodynamic-based scales of Impaired Personality Structure (IPS) was also assessed in parent ratings using the *OPD*-*CA2*-*SQ PR*. This mirrors the findings with adolescent self-report [49] and highlights the close relationship between both diagnostic concepts, OPD Structure and ICD-11 or AMPD Criterion A, in defining the basic impairments concerning PD. Likewise, the strong correlations with most parent-rated DSM-5 AMPD *PID*-*5* maladaptive traits representing Criterion B (except Anankastia) are similar to results obtained with adolescents [74]. This supports the applicability of both Criterion A and Criterion B to assess impairment in personality development, even at younger ages. The complex relationship between the two constructs, Criterion A and B, and the potential incremental validity of one of these constructs over the other should be further investigated in future research. It is important to consider both impaired personality development in childhood, especially in the light of ICD-11 guidelines for diagnosing PD, where type description by maladaptive traits is not mandatory. 

For research purposes, short assessment tools are desirable. Thus, a short version of the self-report inventory named *LoPF*-*Q 12-18 Short* was empirically derived on a trial basis in the current study, strictly parallel to the *LoPF*-*Q 6-18 PR*, also consisting of 36 items and providing the same scales and subscales. This short version showed promising results with equivalent result patterns concerning reliability, factorial validity, and clinical validity compared to the original version of *LoPF*-*Q 12-18* with 97 items. However, the reduction in the number of items is inevitably accompanied by a loss of information. Accordingly, the first evaluation of clinical validity showed clearly lower effect sizes (*d* = 1.1) compared to the original version (*d* = 2.1 standard deviations [49]). Moreover, using the short self-report version, informant agreement with the parent version was lower (0.37 vs. 0.47 for the total scale). This new short version must be regarded as preliminary and will be assessed and further investigated in the EARLY study in several languages (see below). Compared to the *LoPF*-*Q 12-18 Screener* with 20 items [50], the *LoPF*-*Q 12-18 Short* additionally provides the subscale level. 

The domains of IPF, specifically Identity diffusion, have been therapeutically successful in treating adolescent PD. Intensive manualized treatments of young people with Borderline PD achieved significant improvements in personality functioning and 70% remission of cases [12]. This gives hope that the detection of IPF already in childhood in parent-report, e.g., using *LoPF*-*Q 6-18 PR*, sharpens the eye for early signs of relevant personality difficulties or PD. It may lead to the development of early therapeutic interventions tailored for the age 6–11 in order to prevent the development of severe impairments and a full-blown PD and decrease the risk of associated negative effects for the life course of the patients and their families [13,49]. A possible basis for age-specific treatments could be the adaptation of existing therapeutic interventions for adolescents, such as DBT-A or AIT [12]. Alternatively, existing therapeutic approaches for primary school students ages 6–11 could also be adapted to cover specific impairments in personality functioning [75]. Following Herpertz and Bertsch [76], considering the different domains of personality functioning can be useful for treatment formulation and communication with patients and provides new opportunities for individualized, modular treatments focusing on specific impairments and strengths. Very optimistically speaking, this could have the following political implications: early detection could lead to a reduction in PD cases and thus save costs to society if problematic courses can be interrupted early [13]. On the other hand, there could be costs for society if costs arise from the assignment of a PD diagnosis in childhood and assigning special therapies. However, the result of a questionnaire can never be a sufficient basis for the diagnosis of PD. Even if a high level of impairment is indicated through the parent report, detailed clinical interviews and assessments should follow, especially for very young individuals.

### Limitations

The current study has several limitations. First, no definite statement about the mental health status related to the risk of PD in the school sample can be made because no assessment tool was available in German to capture this construct in parent reports for children from 6 years up. Developing a German parent version of the *BPFSP-11* (Borderline Personality Features Scale [77]) is in progress to address this. Second, the sample sizes of the PD subgroups are small (except for Antisocial PD), and the ratio of PD types is atypical, with many Antisocial PD patients. This was due to the fact that many participants in this study were recruited at a forensic department, which might have affected the results. However, results for the clinical validity of *LoPF*-*Q 6-18 PR* were excellent and in the same range as those obtained in a former study on adolescent PD patients (with 44.7% Borderline PD cases) [49]. Further research with larger PD samples, including different types of PD in a balanced way, is needed to investigate potential differences and ensure that the results apply to all PD types. Third, due to the COVID-19 pandemic, there was a contact restriction in the schools throughout the whole study phase. Therefore, the purpose and design of the study could not be explained by the study team as planned in school meetings. Instead, the study procedures were explained by teachers and coaches. This might have affected the participation rate. However, sample descriptives indicate a sufficiently representative school sample considering the ratio of gender, age, and mental health according to *CBCL 4-18*. Fourth, including young adults older than 18 is a limitation, as the assessment tools are not normed for that age range. However, this only applied to *N* = 9 participants. Another limitation is that it is unclear if impairment in personality functioning in childhood, as assessed with the *LoPF*-*Q 6-18 PR*, serves as a valid precursor of later PD development. This has to be investigated longitudinally. In the EARLY-Study starting in 2023, the developmental course of IPF from childhood to adulthood and the relationship between general psychopathology, maladaptive trait expression, and IPF will be investigated in a multi-center setting (EARLY Study—Investigating early signs, risk factors, and the developmental course of Impaired Personality Functioning in Young People. https://brc.ch/research/early/ (accessed on 7 July 2023)). Lastly, this study has been conducted in Europe, and the generalizability of these results to other ethnicities and geographic areas is limited. To address this, several cultural adaptions are in progress (Turkish, French, Spanish, Swahili, Romanian, Lithuanian, Slovenian, and Russian) and will be used in the EARLY-Study for cross-cultural comparisons.

## 5. Conclusions

Altogether, the results speak for the high relevance of the constructs assessed with the *LoPF*-*Q 6-18 PR* to describe impairments associated with PD pathology in adolescents aged 12-18+ and the severity of psychopathology in children aged 6–11. The parent report can be seen as sufficiently equivalent to the self-report measure, showing similar result patterns concerning scale reliability, factorial structure, congruent and discriminant validity, and especially concerning covariation with PD pathology and other measures of psychopathology. Given these results, the *LoPF*-*Q 6-18 PR* can be recommended for the following purposes: (a) Further research concerning the dimensional PD severity approach, especially for longitudinal approaches, as assessments can now start already in childhood and a change of concept is not necessary when reaching adolescence and early adulthood because of the established version *LoPF*-*Q 12-18* for self-report. (b) Diagnostic purposes by assessing Impaired Personality Functioning (IPF) in children and adolescents using parent-report. This supports the involvement of parents in the diagnostic process and a multi-informant approach by using a time-efficient assessment tool with only 36 items. Based on the diagnostic interpretation recommended for young people, a significantly above-average level of impairment (T values over 70) could indicate problematic development in children and lead to detailed clinical assessments in order to improve the early detection of individuals at risk for developing PD. In addition, an individual’s impairment profile in the four different areas of personality functioning could inform treatment or supportive focus and help avoid problematic development.

## Figures and Tables

**Table 1:** Characteristics of the sample.

	School Sample	Patient Sample	Full Sample
*N*	343	256	599
Gender in % m/f	47.1/52.9	47.8/52.2	47.4/52.6
Age	6–21 (*M* 11.1, *SD* 3.6)	6–26 (*M* 13.8, *SD* 3.0)	6–26 (*M* 12.3, *SD* 3.6)
6–11	62% (*N* = 213)	22% (*N* = 57)	45% (*N* = 270)
12–18+	38% (*N* = 130)	78% (*N* = 199)	55% (*N* = 329)
Diagnoses/Status	CBCL T-scores > 70 Internalizing: 5.3% Externalizing: 3.0% Total: 3.0%	Diagnose groups *N* = 38 PD *N* = 70 Comorbid *N* = 88 Internalizing *N* = 38 Externalizing *N* = 22 Other diagnose *N* = 218 No PD	PD types *N* = 18 Antisocial *N* = 8 Borderline *N* = 3 Narcissistic *N* = 2 Anxious-avoidant *N* = 1 Histrionic *N* = 1 Paranoid *N* = 1 Schizoid *N* = 4 Other/not otherwise specified
Rated by	Parents (100%)	Parents (79%) Nurse (18%)	Parents (92%) Nurse (8%)
Gender in % m/f	21.3/78.7	27.6/72.4	23.6/76.4
Age	25-68 (*M* 45.3, *SD* 6.0)	24-73 (*M* 44.9, *SD* 9.1)	24-73 (*M* 45.1, *SD* 7.3)

**Table 2:** Scale reliabilities Cronbach’s Alpha (α) and McDonald’s Omega hierarchical (ωh) of the LoPF-Q 6-18 PR in the full sample *N* = 599 and correlation with the self-report LoPF-Q 12-18 (*N* = 298 school and clinic sample).

Scale	No Items	r-it Range	Reliability α	Reliability ωh	Correlation with Self-Report LoPF-Q 12-18				
					Total	ID	SD	EMP	INT
LoPF-Q 6-18 PR: IPF total score	36	0.49–0.77	0.96	0.99	** 0.47 **	0.45	0.42	0.40	0.45
IPF1 Identity	9	0.46–0.73	0.87	0.96	0.44	** 0.43 **	0.41	0.37	0.40
1.1 Continuity	4	0.40–0.56	0.68	0.74	0.41	0.38	0.38	0.35	0.36
1.2 Coherence	5	0.55–0.70	0.84	0.88	0.40	0.39	0.36	0.34	0.36
IPF2 Self-direction	9	0.55–0.76	0.90	0.94	0.50	0.47	** 0.47 **	0.37	0.47
2.1 Self-Congruence	4	0.47–0.74	0.81	0.91	0.53	0.51	0.50	0.35	0.52
2.2 Purposefulness	5	0.59–0.70	0.84	0.84	0.42	0.39	0.39	0.35	0.38
IPF3 Empathy	9	0.54–0.77	0.90	0.88	0.31	0.27	0.25	** 0.36 **	0.25
3.1 Perspective taking	4	0.56–0.74	0.84	0.87	0.32	0.29	0.26	0.34	0.28
3.2 Prosociality	5	0.51–0.68	0.80	0.84	0.27	0.24	0.21	0.36	0.19
IPF4 Intimacy	9	0.49–0.76	0.88	0.90	0.46	0.44	0.40	0.34	** 0.50 **
4.1 Close relationships	4	0.55–0.70	0.82	0.84	0.41	0.40	0.36	0.29	0.44
4.2 Reciprocity	5	0.44–0.64	0.77	0.83	0.45	0.42	0.39	0.35	0.49

IPF = Impaired Personality Functioning.

**Table 3:** Mean scores (*M*), standard deviation (*SD*), significance (*p*), and effect size (*d*) of *LoPF-Q 6-18 PR* scale differences between age 12–18 general population (*N* = 131) and a subsample of age 12–18 diagnosed PD patients (*N* = 38).

	Sample				Clinical Validity	
	School *N* = 131		PD Patients *N* = 38 Age 12–18		*p*	*d*
	*M*	*SD*	*M*	*SD*		
LoPF-Q 6-18 PR: IPF total score	23.8	14.7	70.3	21.5	<0.001	2.8
IPF1 Identity	4.2	3.1	16.2	6.0	<0.001	3.1
IPF2 Self-direction	7.0	5.2	19.2	7.3	<0.001	2.1
IPF3 Empathy	5.9	4.7	16.9	7.5	<0.001	2.0
IPF4 Intimacy	6.7	4.9	18.0	6.4	<0.001	2.1

IPF = Impaired Personality Functioning; effect size: *d* > 0.20 small, >0.50 medium, >0.80 large.

**Table 4:** Mean scores (*M*), standard deviation (*SD*), significance (*p*), and effect size (*d*) of *LoPF-Q 6-18 PR* scale differences between age 6–11 general population (*N* = 213) and a subsample of age 6–11 patients with mixed diagnoses (*N* = 57).

	Sample				Clinical Validity	
	School *N* = 213		Patients *N* = 57 Age 6–11		*p*	*d*
	*M*	*SD*	*M*	*SD*		
LoPF-Q 6-18 PR: IPF total score	25.5	17.2	68.4	26.0	<0.001	2.2
IPF1 Identity	4.3	3.8	13.8	7.2	<0.001	2.0
IPF2 Self-direction	8.6	6.2	20.3	7.6	<0.001	1.8
IPF3 Empathy	7.0	5.2	18.8	8.1	<0.001	2.0
IPF4 Intimacy	5.6	4.9	15.5	6.6	<0.001	1.9

IPF = Impaired Personality Functioning; effect size: *d* > 0.20 small, >0.50 medium, >0.80 large.

**Table 5:** Correlations of the *LoPF-Q 6-18 PR* scales with the *STiP-5.1* (*N* = 53 clinic samples in Basel), the *PIDBF+ M CA IRF* (*N* = 504 school and clinic samples), the *OPD-CA2-SQ PR* (*N* = 598 school and clinic samples), and the *CBCL 4-18* (*N* = 368 school and clinic samples).

Scale	LoPF-Q 6-18 PR				
	IPF Total Score	IPF1 Identity	IPF2 Self-direction	IPF3 Empathy	IPF4 Intimacy
STiP-5.1					
Total score	0.34 *	0.32 *	0.44 ***	0.09	0.26
Identity	0.47 ***	0.43 **	0.50 ***	0.24	0.41
Self-direction	0.22	0.27 *	0.35 **	−0.02	0.14
Empathy	0.02	0.04	0.09	−0.05	0.00
Intimacy	0.30 *	0.23	0.41 **	0.12	** 0.24 **
OPD-CA2-SQ PR					
Total score	0.96 ***	0.88 ***	0.90 ***	0.87 ***	0.84 ***
IPS2 Identity	0.94 ***	0.86 ***	0.94 ***	0.80 ***	0.82 ***
IPS1 Control	0.83 ***	0.79 ***	0.75 ***	0.84 ***	0.66 ***
IPS3 Interpersonality	0.90 ***	0.81 ***	0.82 ***	0.81 ***	0.85 ***
IPS4 Attachment	0.86 ***	0.80 ***	0.78 ***	0.76 ***	0.78 ***
PID5BF+ M CA IRF					
Negative Affectivity	0.62 ***	0.56 ***	0.66 ***	0.54 ***	0.50 ***
Detachment	0.78 ***	0.70 ***	0.71 ***	0.64 ***	0.81 ***
Antagonism	0.65 ***	0.64 ***	0.50 ***	0.74 ***	0.50 ***
Disinhibition	0.66 ***	0.62 ***	0.64 ***	0.69 ***	0.47 ***
Anankastia	0.25 ***	0.23 ***	0.20 ***	0.19 ***	0.28 ***
Psychoticism	0.69 ***	0.66 ***	0.60 ***	0.67 ***	0.58 ***
CBCL 4-18					
Total score	0.78 ***	0.72 ***	0.72 ***	0.73 ***	0.60 ***
Internalizing	0.70 ***	0.62 ***	0.65 ***	0.55 ***	0.66 ***
Externalizing	0.68 ***	0.63 ***	0.58 ***	0.74 ***	0.44 ***

IPF = Impaired Personality Functioning; IPS: Impaired Personality Structure; Significance *p* * = 5%, ** = 1%, *** = 0.1% level; effect size: r > 0.10 small, >0.30 medium, >0.50 strong.

## Data Availability

Data are unavailable due to privacy restrictions.
